# Flipped learning enhances non-technical skill performance in simulation-based education: a randomised controlled trial

**DOI:** 10.1186/s12909-021-02766-w

**Published:** 2021-06-22

**Authors:** Parisa Moll-Khosrawi, Christian Zöllner, Nadine Cencin, Leonie Schulte-Uentrop

**Affiliations:** Department of Anaesthesiology, University Medical Centre Hamburg-Eppendorf, Martinistr. 52, 20246 Hamburg, Germany

## Abstract

**Background:**

Many efforts of the past years aimed to build a safer health care system and hereby, non-technical skills (NTS) have been recognised to be responsible for over 70 % of preventable medical mishaps. In order to counteract those mishaps, several simulation-based trainings have been implemented in health care education to convey NTS. Still, the best and effective way to foster NTS in simulation-based training is not known. Due to the importance of NTS, this gap in knowledge needs to be filled. A possible approach to convey NTS effectively during simulation-based medical education (SBME), might be the use of the flipped learning approach. The benefits of flipped learning regarding the improvement of human factors (NTS), have not been investigated yet. Therefore, the authors introduced flipped learning as an experimental intervention into their SBME emergency trainings and aimed to analyse, whether flipped learning improved students´ NTS performance compared to lecture-based learning (LBL).

**Methods:**

In a randomized controlled trial, 3rd year medical students participated in a SBME training and then received either a further SBME training with integrated flipped learning on NTS (intervention), or a further SBME training and an accompanying lecture on NTS (control). NTS performance was assessed on three skill dimensions with a validated behavioural marker system.

**Results:**

The authors analysed NTS performance of 102 students, prior and after their allocation to each teaching method. The baseline NTS performance of both groups did not differ, whereas the intervention group enhanced significantly on all three skill dimensions (*t* (44) = 5.63, *p* < .001; *t* (44) = 4.47, *p* < .001; *t* (44) = 4.94, *p* < .001).

**Conclusion:**

The integration of flipped learning into SBME yields a significant improvement of NTS performance and therefore medical educators should consider the application of flipped learning to convey complex human factors and skills.

## Introduction

Since “The Institute of Medicine” published over 20 years ago the landmark report “To err is human”, many efforts have been done to make healthcare safer and reduce preventable mishaps [1]. In this context, human factors (non-technical skills) have been recognised to be responsible for over 70 % of medical mishaps [2, 3]. Therefore, high quality medical care requires technical skills (TS) alongside with non-technical skills (NTS) [4–7]. TS refer to medical knowledge and practical procedures (like chest compression, inserting an intravenous line), whereas NTS are defined as “the cognitive, social and personal resource skills that complement technical skills, and contribute to safe and efficient task performance”[7].

Training of NTS should, as recommended by several position papers, should take place as early as possible in health care education [8]. For this purpose, the WHO has even published a safety curriculum guide for medical schools [9]. The emphasis on the importance of early implementation of NTS training is based on the knowledge that NTS are not acquired through clinical practise and routine [10]. Therefore, many medical faculties have adapted their curricula and implemented simulation-based trainings to address NTS in undergraduate education [11].

Although simulation-based medical education (SBME) has been recognised as the ideal instructional design to train NTS, the best and effective way to teach and train NTS during SBME is not known [12, 13].

A possible approach to convey NTS effectively during SBME might be the use of the flipped learning approach, which has been adopted into various undergraduate and postgraduate healthcare curricula [14, 15]. Flipped Learning is an instructional approach in which the traditional concept and idea of classroom-based learning is inverted [16]. New learning contents are first mastered with structured activities in the individual learning space. The face-to-face time (group learning space) is used to accelerate the learning cycle by using more active learning strategies [17]. Therefore, according to the learning cycle model [18, 19], the active content attainment takes place in the individual learning space and the concept application shifts to the class time [16, 20–22]. To promote the reflective process and enhance learning achievements, the provision of an adequate learning guidance is important for the individual learning phase. Therefore, flipped learning needs proper planning and organisation and the concept consists not only of the distribution of additional tasks [23, 24]. Flipping a class, like having learners read additional material outside the class, does not necessarily result in flipped learning [16].

The acquisition and the transfer of NTS into behavioural patterns is a complex process which is often not achieved with diligence and learning efforts- it requires deep learning, reflection and the creation of mental models [25, 26]. Therefore, the elements “flexible environment” and the “intentional content” of the flipped learning approach could contribute to enhanced learning achievements of NTS: Students are enabled to control their speed of learning, to reflect as often as possible on the learning contents and hereby activate deep processing, build mental models and develop targets for action plans to improve performance [27].

As suggested by Chen and colleagues in a systematic review [15], as well as in an investigation of Tang and colleagues in ophthalmology teaching [28], one positive side-effect of flipped learning could be enhanced (autonomous) motivation. This phenomenon occurs due to autonomy supported and learner-oriented learning, which results in an enforced identification process with the learning contents [29]. A brief insight into motivational theories explains how this desirable side-effect might come about: The Self-determination theory of motivation describes different motivational qualities that underlie human behaviour (behavioural regulation). When motivation derives from heartfelt interest or personal endorsement, a person is autonomously motivated. Autonomous motivation is composed of the motivational qualities “intrinsic” and “identified “. The opposite concept is controlled motivation (“external”- and “introjected regulation”). Here, motivation derives from external- or internal pressure which is formed by desires for bounty [30]. To support autonomous motivation, three basic psychological needs have to be satisfied: Autonomy, competence and relatedness [31]. Several studies have confirmed autonomous motivation as the type of motivation that leads to better well-being, better learning and greater academic success [32, 33]. The flipped learning approach would help the students to reflect on the importance of non-technical skills during the individual learning space, other than just except them because they are part of their curriculum learning contents. This reflection process shifts the locus of causality (the “why” of engaging in an activity) from the outside (curriculum learning goals) to the inside (identification with the task) [31]. The process of re-location leads to enhanced autonomous motivation, which has been repeatedly emphasized by curriculum developers, to be an important determinant of academic success and learning [32]. Therefore, flipped learning could be one approach to enhance the (desired and demanded) dependent variable of students´ motivation.

Although several studies suggest that the flipped approach improves learning in healthcare education and is superior compared to classical learning approaches [15, 20, 22, 34], to our best knowledge, no published study has investigated if flipped learning yields significant improvements in human factors- like NTS performance.

Therefore, we introduced flipped learning as an experimental intervention into our SBME emergency trainings and aimed to analyse whether flipped learning improved students' NTS performance. Furthermore, we compared the SBME and flipped learning approach with SBME and lecture-based learning (LBL) with respect to NTS performance. We also explored whether the flipped learning approach had an effect on the affectional dimension of learning and analysed students' motivation of both study groups, towards participating in the SBME teaching approaches. We hypothesised, that the flipped learning yielded an improvement of NTS performance itself and compared to the lecture-based conventional training (primary outcome measure). The secondary outcome was students´ motivation to participate in the SBME.

## Methods

 This prospective randomised controlled simulation study with blinded participants was performed at the University Medical Center Hamburg-Eppendorf during Winter semester 2019/20.

### Participants

To reduce performance bias, 3rd year medical students were chosen as study participants, because they were familiar with simulation trainings.

The students were divided into subgroups and the subgroups were randomized to the intervention- or control group (computer-generated random numbers [35]). To prevent a Hawthorne effect (modification of behaviour due to the knowledge of observation), the students did not know to which group they were randomized to (blinded participants) and they did not know when and what was scored during the trainings [36]. Each group participated in two trainings (first training was for the assessment of the baseline). The trainings were scheduled within one week (Tuesday and Thursday) of the study period for each subgroup.

 Participation in the study was voluntary and written informed consent was attained from every study participant.

All students were asked to keep discreet about the details of their training and not to enclose it to their fellow students.

### Study setting

After the first training (baseline), the groups received two different teaching approaches on NTS according to their allocation (flipped learning or lecture-based learning) and a further SBME training.

#### Baseline training

A maximum of 12 students participated per training and the duration of each training was 150 min. The two groups of students were supervised by the same medical teachers (instructor), who were experienced physicians of the department of anaesthesiology with extensive knowledge and training in emergency medicine, cardiac life support, medical education and rating NTS during simulation trainings. The learning objectives of each training were accessible through an online platform of the medical faculty.

The first simulation training of both groups had the same structure and the same procedure and was a conventional SBME training. Likewise, the same simulation scenarios were carried out in both groups with the same learning objectives.

At the beginning of each training a 30-minute seminar was held to refresh theoretical knowledge of emergency medicine and cardiac life support. Principles of Crew Resource Management (CRM) were addressed briefly, using examples from aviation.

Each training consisted of three standardized assigned simulation scenarios (emergency scenario with cardiac arrest) and each scenario was conducted in a different room of the simulation centre of the department of anaesthesiology, using high fidelity manikins (Resusci Anne, Laerdal Medical AS, Stavanger, Norway). The participants of each training (pre-defined subgroups of students by the dean´s office- maximum of 12 students per training) were divided into smaller subgroups (5 to 6 students per subgroup) to rotate through the three scenarios and one instructor supervised each scenario. From these smaller subgroups three undergraduates were assigned randomly to execute the scenario. The assignment of the roles for each scenario (one team leader (physician) and two nurses/paramedics) was also randomly. The remaining students of the smaller subgroup participated as neutral observers.

A debriefing was conducted after each scenario, focusing on three conceptual phases: gathering, analysing, summarising. The debriefing was held in the conventional way and the role of the instructor (medical teacher) during the debriefing was that of a teacher.

### Teaching methods of NTS: Interventional and control training

#### Flipped teaching method (intervention)

##### Preparation phase

Six months prior to the study period, three medical teachers were engaged in the preparatory phase of the flipped learning approach. According to the backward instructional design, first learning goals (NTS) were identified and then opportunities for pre-learning were discussed and active learning strategies were specified. Afterwards the final design of the flipped learning was determined, which included the four pillars of F-L-I-P ™, as described by the “Flipped Learning Network” (**F**lexible environment, **L**earning culture, **I**ntentional content, **P**rofessional educator) [37].

The contents which were carried out in flipped learning were the definition and explanation of NTS with reliance to the AS-NTS rating system and relevant benchmarks, which took mostly place in the individual learning space. The students were given the task to prepare themselves in the best possible way on NTS and AS-NTS and its benchmarks, in order to carry out the debriefing in the upcoming training. This step was designed to foster reflection on NTS prior to the second training.

To facilitate learner-centered environment, all the teaching activities were designed based on instructional scaffolding:

Pre-training (class) contents.

- *Brief seminar on NTS held at the end of the baseline training (twenty minutes)* This seminar included following contents: History of how NTS were translated from aviation to healthcare. An introduction to the rating system Anaesthesiology Students´ NTS (AS-NTS) [11] with explanatory behavioural benchmarks for each dimension of the AS-NTS. For this purpose, scenarios and situations were talked through with focus on how good NTS performance would be like.
- At the end of this seminar *teaching materials were provided*, which included a script on NTS and behavioural benchmarks, a PowerPoint courseware and a detailed description about how AS-NTS was developed and how its application works.
- *Individual learning space (instructional activities)*: A set of clear defined work assignments, which included to work through the provided teaching materials; to complete a written debriefing of the three scenarios which the students had observed in the baseline training (*reflection, cold written debriefing*)- hereby the application and description and definition of fitting behavioural benchmarks (targets for action) were encouraged (NTS score based on the AS-NTS).

Furthermore, the students were given the assignment to get as familiar as possible with the AS-NTS, because it would be their responsibility to conduct the debriefings of the following training and to provide an AS-NTS score for each scenario.

Classroom phase (*group learning space*), second training.

- At the beginning of the training, outstanding questions were clarified and students were asked if every assignment of the individual learning were completed and if they could define behavioural benchmarks. Then, similar to the first training, the undergraduates were divided into smaller groups (randomly) to rotate through three simulation scenarios. The small groups remained together for all three scenarios. Each scenario and its debriefing had the duration of thirty minutes.
- The debriefings of each scenario were conducted by the observing students of each small group in terms of collaborative learning by team based learning: Three students conducted the scenario as physician and nurses/paramedics. The observing students (two/three) filled out an AS-NTS score while watching the scenario and took notes. The scenarios contained cardio-pulmonary emergencies, like acute coronary syndrome. After each scenario, the observing students had five minutes to discuss their debriefing in a different room, based on their AS-NTS score. Then they provided the debriefing for their peer-students. The role of the instructors was to act like a facilitator to provide a good learning environment and encourage participation.

The provision of the AS-NTS score and debriefing by the students was not for the outcome analysis. The purpose of this exercise was to enable the students to reflect on NTS and apply what they had prepared in their individual learning space. Therefore, the instructors filled out the AS-NTS as well and complemented the peer-debriefing, because the analysis of NTS performance was based on the instructor ratings.

Eight trainings were conducted as pilots to train the instructors, to close remaining gaps, to standardise the intervention and to detect necessary changes of the study design and to identify an effect size for the sample size calculation.

##### Lecture-based learning method (control group)

Pre-training (class) contents.

- After the baseline training, the control group received a 90-minute lecture on NTS called “To err is human”. This lecture addressed the same learning objectives which were conveyed to the intervention group and was held by a professor of anaesthesiology and didactics whose lectures were always evaluated as outstanding.

Classroom phase (*group learning space*), second training.

**Fig. 1:**
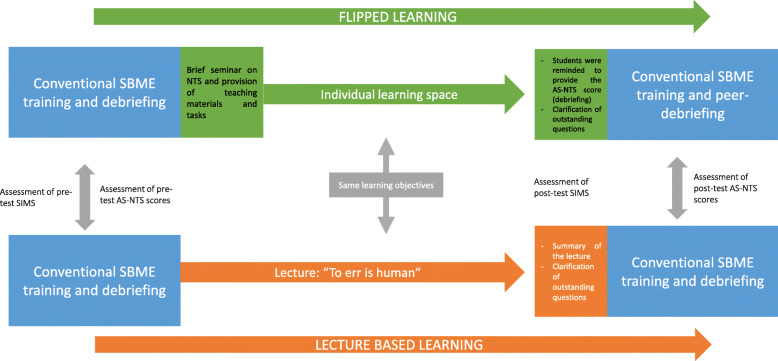
Depicts the research flow and procedures

- At the beginning of the training, remaining questions were clarified and a brief summary on NTS and the contents of the lecture were given. Then, similar to the first training, the undergraduates were divided into smaller groups to rotate through three simulation scenarios.
- After each scenario a debriefing was held by the instructors, based on the AS-NTS scoring (Fig. 1).

Table 1. compares the two different teaching approaches in the context of our study design.

**Table 1:** Comparison and differences of the teaching approaches

	Flipped Learning	Lecture Based Learning
*Learning goals (NTS)*	Described on an online platform	Described on an online platform
*First training*	Conventional simulation-based training with instructor-led debriefing	Conventional simulation-based training with instructor-led debriefing
*Material/Notes*	Outside of class	In class (lecture)
*Teacher´s role*	Facilitator (introduction to the learning contents and assignment of tasks)	Teacher (lecture)
*Student involvement*	High (tasks of the individual learning space)	Relatively low (active or passive listening during lecture)
*Introduction to NTS*	Brief interactive seminar (end of the first training)	Lecture
*Description of NTS*	Individual learning space (learning material was provided and precise tasks were given)	Lecture
*AS-NTS taxonomy*	Brief interactive seminar; individual learning space	Lecture
*Behavioural benchmarks for different NTS*	Individual learning space: Brief cold (written) debriefing on the preceding training scenarios	Lecture
*Before second training*	Open questions were clarified to ensure preparation (interactive)	Summary by teacher
*Role of students in the second training*	Students were given the task to prepare themselves in order to provide the debriefing	None

*NTS* Non-technical skills; *AS-NTS *Anaesthesiology students´ non-technical skills.

### Measure tools

#### Primary outcome measure: Performance of NTS

The German version of “Anaesthesiology students´ Non-Technical skills” (AS-NTS) [11] was used by the instructors to rate NTS. AS-NTS is composed of three dimensions:

1. Planning tasks, prioritising and problem-solving.
2. Teamwork and leadership.
3. Team orientation..

Performance is rated on a five-point Likert scale *(1 = very good; 5 = very poor).* An underlying skill structure is used to give behaviourally anchored rating examples to clarify what a “good” or “poor” performance on each dimension might look like.

Validity, feasibility and sufficient coverage of relevant NTS in the AS-NTS have been previously reported [11].

#### Secondary outcome measure: Situational Motivation to participate in the trainings

Motivation was measured with the German version of the “Situational Motivation Scale” (SIMS), which measures the underlying motivation to participate in a task or activity at a specific point of time (situational) [38]. The SIMS was developed based on the Self-determination theory (SDT) of motivation [29].

Four subscales (intrinsic motivation, identified- introjected- external regulation and amotivation) are assessed with 20 items. Each item has a 7-point Likert scale (*1 = Does not correspond at all; 7 = Corresponds exactly*).

Autonomous motivation is computed by adding and averaging intrinsic motivation and identified regulation. Controlled regulation is computed by adding and averaging external- and introjected regulation [38, 39].

Reliability and validity of the SIMS, as well as the German translation has been confirmed in many studies [39, 40].

The undergraduates filled out a paper-based SIMS questionnaire at the beginning of the first (pre-test)- and at the beginning of the second (post-test) training.

### Statistical analysis

Statistical analysis was performed using IBM SPSS Statistics Version 23.0.

The sample size was calculated based on the effect size (partial *η²* dimension one: 0.21, dimension two 0.5, dimension three 0.36) which was calculated from the results of the pilot study. A sample size of totally 64 study participants were necessary with a two-sided 5 % significance level and a power of 90 %.

Descriptive statistics were used for the calculation of mean values of each AS-NTS dimension and for computing the subscale scores of the SIMS.

For the primary outcome, first a paired t-test was calculated, followed by a factorial ANOVA (2 way)- to compare the main effects of “Time” (independent variable, within-group, 2 levels) and “Group” (independent variable, between-group, 2 levels) as well as their interaction effects on the AS-NTS scores. Homogeneity of error variances in each group, as assessed by Levene´s test (*p* > .05) was given.

The dependent variables were the scores of each AS-NTS dimension.

Model-estimated marginal means with 95 % confidence intervals were computed and Bonferroni adjusted pairwise comparisons were conducted. Post-hoc tests were then calculated for planned comparison and exploration of the effects.

For the secondary outcome a paired t-test was calculated. The assumptions of a paired t-test were not violated by our data. There were no outliners in the data and the differences between the pre- and post-test scores were normally distributed as assessed by the Shapiro Wilk test.

## Results

### Participants

**Fig. 2:**
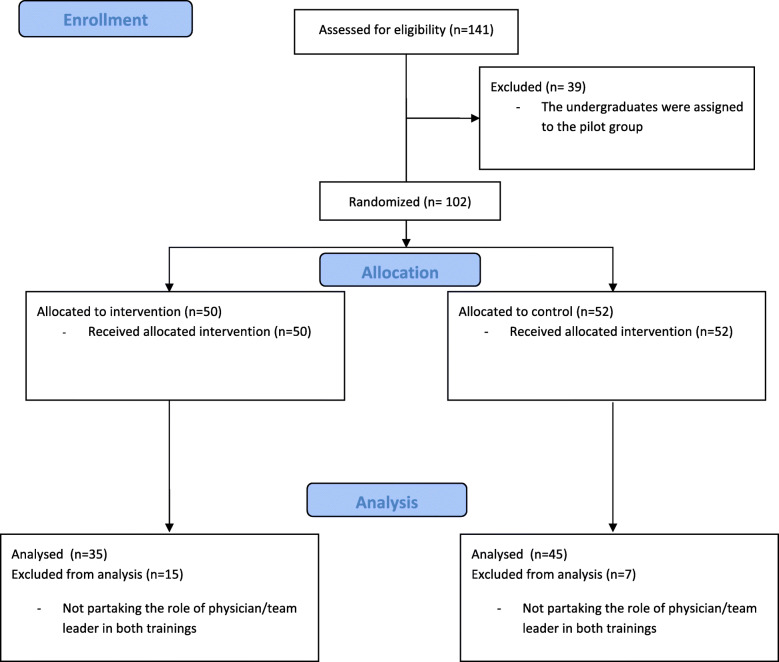
Depicts a participant flow diagram including all steps of the study.

After the pilot phase, a total of 102 3rd year medical students were included in the study. Inclusion criteria was the take on of the physician´s role in both trainings, because data was analysed continuously with repeated measures. Therefore, 22 of 102 students had to be excluded from the final analysis because due to the size of the training subgroups, they did not take on the physician´s role in both trainings (Fig. 2).

The demographics of the randomised and analysed undergraduates were not significantly different (Table 2).

Nine instructors conducted the trainings for the interventional and for the control group.

**Table 2:** Demographic data of the study participants

	*Intervention group* *n = 35*	*Control group* *n = 45*
Age (years) Sex (female) % (n) Sex (male) % (n) Additional CPR-and or/ emergency training (despise the trainings of the curriculum) Prior medical knowledge or medical work experience (training as paramedic, nurse etc.)	22.8 48.6 (17) 51.4 (18) None None	22.9 53.3 (24) 46.7 (21) None None

### Outcomes

#### Primary outcome measure: Performance of NTS

As shown in Table 3., NTS performance, assessed with the AS-NTS rating score, were high on all three dimensions in both groups (this complies with low numeric scores of AS-NTS: 1 = very good-, 5 = bad performance).

The results confirmed our hypothesis, that the flipped learning approach enhances NTS performance: The AS-NTS scores of the intervention group enhanced significantly after the intervention on all dimensions (dimension one: *t* (44) = 5.63, *p* < .001; dimension two: *t* (44) = 4.47, *p* < .001; dimension three: *t* (44) = 4.94, *p* < .001).

**Table 3:** NTS performance (pre- and post-test) assessed with the AS-NTS

*Intervention group* *n = 35*							*Control group* *n = 45*					
	*Pre-Test* *M SD*	*Post-Test* *M SD*	*Mean* *difference*	*95 % CI*		*p*	*Pre-Test* *M SD*	*Post-Test* *M SD*	*Mean* *difference*	*95 % CI*		*p*
				*LL*	*UL*					*LL*	*UL*	
D1	2.53 0.89	1.84 0.82	0.69*	0.44	0.94	< 0.001	2.20 0.96	1.91 0.82	0.29	− 0.11	0.68	0.152
D2	2.38 0.86	1.71 0.60	0.68*	0.37	0.97	< 0.001	2.20 0.76	2.03 0.66	0.17	0.50	− 0.17	0.312
D3	2.36 0.80	1.69 0.59	0.68*	0.4	0.94	< 0.001	2.20 0.63	2.03 0.75	0.17	− 0.11	0.45	0.226

Note: The pre-test scores are the baseline scores, assessed in the first training of both groups. The post-test scores were assessed in the second training (which was after the intervention in the intervention group). * The differences were significant at *p* < .001.*M* Mean value. *SD* Standard deviation, *D1* Dimension one of AS-NTS “Planning tasks, prioritising and problem-solving”, *D2* Dimension two of AS-NTS “Teamwork and leadership”, *D3* Dimension three of AS-NTS “Team orientation”. *CI *confidence interval; *LL* lower limit; *UL* upper limit

Correlation analysis between the variables indicated that the dependent variables (dimensions of AS-NTS) are not independent: τ _*b*_ (dimension one and two) = 0.506;

τ _*b*_ (dimension one and three) = 0.394; τ _*b*_ (dimension two and three) = 0.555.

**Fig. 3:**
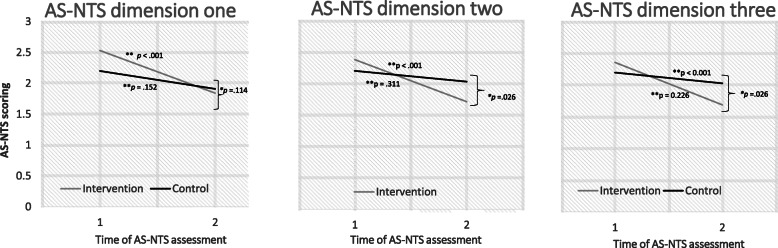
Estimated marginal means of NTS performance of both trainings for each group

The results of the main- and interaction effects of the factorial ANOVA are shown in Table 4; Fig. 3, which also confirmed our first hypothesis (flipped learning enhances NTS) as well our second hypothesis (flipped learning is superior compared to LBL).

**Table 4:** F-ANOVA results: Main and interaction effects

*Effects / Predictor*	*df*_*Num*,_*df*_*Den*_	*F*	*p*	*partial η²*
*AS-NTS dimension one: Planning tasks, prioritising and problem-solving*				
Interaction effect (Time*Group) Main effect “Time” Main effect “Group”	1 ,78 1, 78 1, 78	3.34 19.50 0.65	0.072 < 0.001* 0.421	0.04 0.20 0.01
*AS-NTS dimension two: Teamwork and leadership*				
Interaction effect (Time*Group) Main effect “Time” Main effect “Group”	1, 78 1, 78 1, 78	4.89 14.01 0.34	0.030* < 0.001* 0.561	0.06 0.15 0.004
*AS-NTS dimension three: Team orientation*				
Interaction effect (Time*Group) Main effect “Time” Main effect “Group”	1, 78 1, 78 1, 78	6.39 18.29 0.55	0.014* < 0.001* 0.459	0.08 0.19 0.01

Note: df_Num_ indicates degrees of freedom numerator; df_Den_ indicates degrees of freedom denominator.

* Indicates significant result. A significant effect of “Time” indicates that there were changes in NTS performance over the time, averaged across the whole sample- the significant interaction effect confirms, that these changes time were not equivalent across the two groups. This means that the independent variables “Time” and “Group” had a combined effect on the dependent variable NTS performance on dimension two and three- due to the significant interaction effect, the main effects of “Time” do not need further consideration. There was no significant interaction effect and no significant main effects of “Group” for dimension one. Only the mean effect of “Time” was significant, indicating, that there were changes over time across the whole sample, but with no difference between the study group. *Intervention group n = 35; Control group n = 45.*

As shown in Table 4, there was a significant effect of “time” for all AS-NTS dimensions, this indicates that there were changes in NTS performance over the time, averaged across the whole sample. The follow up of these results indicates that these changes were not equivalent across the two groups: There was no significant difference between the groups at baseline and the control group did not change significantly over time (dimension one: *F* (1, 34) = 2.15, *p* = .152, partial *η²* = 0.06; dimension two: *F* (1,34) = 1.06, *p* = .310, partial *η²* = 0.03; dimension three: *F* (1,34) = 1.52, *p* = .226, partial *η²* = 0.04), whereas the mean scores of the intervention group changed significantly over time (dimension one: *F* (1,44) = 31.70, *p* < .001, partial *η²* = 0.42; dimension two: *F* (1,44) = 20, *p* < .001, partial *η²* = 0.31; dimension three: *F* (1,44) = 24.44, *p* < .001, partial *η²* = 0.36).

The comparison of the post-test NTS performance between the groups showed that the scores of the intervention group were significantly different (better) on dimensions two and three (dimension two: *F* (1, 78) = 5.12, *p = .026;* dimension three *F* (1, 78) = 5.20, *p = .026*) compared to the control group. On dimension one, the post-test scorings of the two groups did not differ significantly (*F* = (1, 78) = 2.56, *p* = .114).

#### Secondary outcome measure: Situational Motivation to participate in the trainings

Autonomous- and controlled motivation, assessed with the SIMS were comparable in both groups: high levels of autonomous- and mediocre to low levels of controlled regulation. In both groups, differences between the pre- and post-test measurements of motivation were negligible and not significant (Table 5).

**Table 5:** Situational Motivation toward participating in the trainings

*Intervention group* *n = 36*									*Control group* *n = 40*							
*Type* *of motivation*	*Pre-Test*		*Post-Test*		*T(36)*	*p*	*95 % CI*		*Pre-Test*		*Post-Test*				*95 % CI*	
	*M*	*SD*	*M*	*SD*			*LL*	*UL*	*M*	*SD*	*M*	*SD*	*T(40)*	*p*	*LL*	*UL*
Autonomous	5.03	0.82	5.07	0.81	− 0.28	0.783	− 0.28	0.21	5.28	0.99	5.07	0.81	1.64	0.109	− 0.75	0.72
Controlled	3.52	1.18	3.38	1.06	0.69	0.495	− 0.28	0.56	3.63	1.07	3.25	1.16	1.44	0.157	− 0.15	0.91

Note: The pre-test SIMS score was assessed prior to the first- and the post-test score prior to the second training (after the intervention for the intervention group). *M* = Mean value; *SD* = Standard deviation. CI = confidence interval; *LL* = lower limit; *UL* = upper limit.

## Discussion

In our randomised controlled trial, we confirmed our hypothesis and found that a flipped learning approach to train NTS enhances significantly NTS performance of 3rd year medical students, compared to a conventional lecture-based approach combined with simulation.

Several studies in medical education have confirmed the positive benefits of flipped learning on students´ learning and knowledge acquisition and proved its superiority over traditional teaching approaches [15, 23, 34]. Our results confirm the previous findings and extend them with advantages of flipped learning regarding the improvement of human factors (NTS), which complies with level 3 of Kirkpatrick´s framework [41].

The improvement of human factors is a challenge in medical education and recently a lack of evidence of how to teach and train NTS has been identified [5, 13]. As NTS are gained through the socialisation process of every individual [42], the learning process of NTS is detached from learning processes of factual knowledge. Factual knowledge can be acquired with diligence and studying, whereas the acquisition of NTS is far more complex and requires a transfer of knowledge and behavioural benchmarks into one´s own behavioural patterns [42]. Therefore, many interventions and investigations failed to find the ideal approach to teach and foster NTS in simulation trainings [5, 25]. These investigations mainly focused on post simulation debriefings [5]. The reasons why NTS were not conveyed effectively in the reported studies, can be explained with principles of learning psychology [43, 44]. The training itself as an isolated instructional design, might not be the ideal setting, as learning does not occur in a closed system, where the instructor provides knowledge and the students simply absorb it [45]. Secondly, students who participated in a simulation scenario, directly prior to the debriefing, might not have the emotional absorption capacity for the debriefing input, leading to cognitive overload and resulting in a declining learning process [44]. Certainly, the post simulation debriefing is a crucial component to promote the actual learning process during SBME [12, 46, 47]. Nevertheless, in order to foster active learning and accelerate learning from experience by promoting reflection during SBME, some concepts and factual knowledge have to be reviewed beyond the boundary of formal class time with self-directed instructional activities [45, 48].

In our study, we created these prerequisites of learning for the students, wherein, among other factors, lies the explanation for our results: First, the students had the opportunity to control the learning speed during the individual learning space. Secondly, the instructional activities were designed to connect prior experience (first training) with information and behavioural benchmarks and hereby fostered the process of reflection, which in turn enabled students to develop their own knowledge and mental models of NTS [17, 45, 49, 50]. The reflection on NTS behavioural benchmarks and the building of mental models during the individual learning space, promoted the learning cycle and resulted in a type of formative assessment [51, 52], which accelerated the transfer of factual knowledge to behaviour. Due to its importance, we supported and facilitated the process of mental modelling by illustrating general benchmarks of NTS (provision of the AS-NTS framework) [11]. It was not the peer-debriefing- but far more the responsibility to carry out the debriefings which fostered the reflective process, enhanced deep learning and the building of mental models during the individual learning space. Hereby the transfer of NTS into behaviour and practise was facilitated. Therefore, the task to prepare for the debriefings, might have been the most decisive part of the individual learn space of the flipped learning approach.

The formation of mental models is important to enhance NTS, as it has been demonstrated that mental models help to enhance team effectiveness and to act properly under varying conditions [26, 52–54]. According to experimental learning, the building of mental models complies with the generalization stage, in which the students think and reflect critically and analyse what might apply in real clinical practise [55]. Finally, this generalization stage lead to the retrieval of the prior built mental models, during the face-to-face time (second training), resulting in enhanced NTS performance.

Thus, it can be summarised, that conveying the complex topic of NTS with the flipped learning approach enhanced students´ learning experience and fostered positive learning outcomes and behavioural changes. The graduated approach enabled the students to have the ownership of their learning process and prevented cognitive overload [17, 56].

One might argue that according to constructive alignment, the theoretical teaching approach which we designed for the individual learning space, might not be appropriate [57]. Considering that the theoretical instructional activities fostered the reflective process and therefore the transfer of knowledge to action, this objection can be overruled. Furthermore, a first theoretical approach seems to be necessary, in order to foster active learning during SBME.

Interestingly, the baseline scorings of the control group were, even if not statistically significant, better than the scores of the intervention group. One can argue that the intervention group enhanced skills more than the control group, because there was more potential for improvement. However, no ceiling effect occurred in the control group, which indicates, that further improvement is still possible.

Curriculum developers have repeatedly emphasised the positive impact of autonomous motivation on learning success and academic achievement in medical students [30, 33]. Therefore, the integration of autonomy supportive instructional designs and teaching approaches into medical education have been recommended [58]. We hypothesised that the flipped learning approach would enhance students´ autonomous motivation due to its learner oriented and autonomy supportive features. Nevertheless, we did not find any increase of students´ autonomous motivation, although- based on SDT- the pillars of autonomous motivation, which comply with the satisfaction of the three basic psychological needs, were supported by the intervention: The teamwork during each simulation scenario had the potential to create the feeling of connection to their other students or peers (relatedness). The conduction of the debriefing was transferred to the students (competence) and hereby they had the autonomy over their own learning (autonomy) [30, 31]. An increase of autonomous motivation means that the locus of causality, the “why” of doing something, relocates from external to internal [31]. The intervention might have been too short to have such effects on students´ motivation.

Teaching and fostering NTS in healthcare education is inevitable to enhance patient safety and to provide best medical care. Therefore, it is the duty of us medical educators to provide curriculum approaches and facilitate the teaching and learning of NTS.

Our study draws a valuable implication for educational practise: We recommend the application of a flipped learning approach in combination with SBME to complement the learning process of NTS. Our suggested approach is easy assessable, easy to implement and cost-effective. The brief seminar of the intervention can be replaced with a video which can be provided via online platforms.

While we paid plenty attention to the design of our study, some limitations provide a new scope for future research. One limitation is that we did not assess the learning styles of the students, which is an important determinant of learning success. As flipped learning and LBL are two different teaching approaches, it would have been interesting to access learning styles of the students and correlate them with their NTS performance and the teaching approach. Then more differentiated conclusions would have been possible to be drawn on the effect of flipped learning on NTS performance and motivation, with respect to students learning characteristics.

Furthermore, our data does not provide information which component of the flipped learning had the greatest effect on skill enhancement. We can only draw the conclusion that the multifactorial concept and the preparation to conduct the debriefing (individual learning space) resulted in enhanced NTS performance- but our results do no clarify if the conduction itself enhanced NTS as well. For this purpose, a further training and assessment would have been necessary. Future studies should explore the effect of peer-debriefing on NTS performance.

A further limitation of our study is that medical educators were not blinded. However, they were highly motivated to support the study as objectively as possible and not to be influenced by the information of students´ allocation. Furthermore, in order to minimize potential bias, they were instructed to finish their AS-NTS ratings before the students provided feedback.

## Conclusions

Flipped learning is an ideal teaching approach to introduce complex teaching contents which include behavioural changes (skills). The combination of flipped learning and SBME leads to NTS enhancement.

## Data Availability

Data will be provided from the corresponding author on reasonable request.
